# Determining the predominant tautomeric structure of iodine-based group-transfer reagents by ^17^O NMR spectroscopy

**DOI:** 10.3762/bjoc.14.203

**Published:** 2018-08-30

**Authors:** Nico Santschi, Cody Ross Pitts, Benson J Jelier, René Verel

**Affiliations:** 1 Eidgenössische Technische Hochschule (ETH) Zürich, Department of Chemistry and Applied Biosciences, Vladimir-Prelog-Weg 1/2, 8093 Zürich, Switzerland

**Keywords:** electrophilic, hypervalent iodine, ^17^O NMR spectroscopy, trifluoromethylation, trifluoromethylthiolation

## Abstract

Cyclic benziodoxole systems have become a premier scaffold for the design of electrophilic transfer reagents. A particularly intriguing aspect is the fundamental I^I^–I^III^ tautomerism about the hypervalent bond, which has led in certain cases to a surprising re-evaluation of the classic hypervalent structure. Thus, through a combination of ^17^O NMR spectroscopy at natural abundance with DFT calculations, we establish a convenient method to provide solution-phase structural insights for this class of ubiquitous reagents. In particular, we confirm that Shen’s revised, electrophilic SCF_3_-transfer reagent also adopts an "acyclic" thioperoxide tautomeric form in solution. After calibration, the approach described herein likely provides a more general and direct method to distinguish between cyclic and acyclic structural features based on a single experimental ^17^O NMR spectrum and a computationally-derived isotropic shift value. Furthermore, we apply this structural elucidation technique to predict the constitution of an electrophilic iodine-based cyano-transfer reagent as an NC–I–O motif and study the acid-mediated activation of Togni's trifluoromethylation reagent.

## Introduction

**Figure 1:**
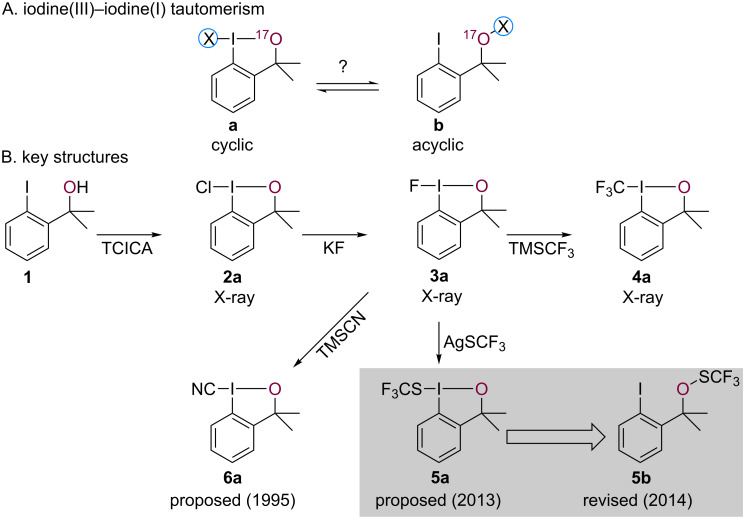
Tautomerism in iodine-based group-transfer reagents probed by ^17^O NMR spectroscopy (A) and key structures investigated herein (B).

The remarkable stability and reactivity of Togni's hypervalent iodine-based trifluoromethylation reagents (e.g., **4a**) [1] have inspired the development of analogous compounds, including a well-known SCF_3_-transfer reagent **5** in 2013 by Shen and co-workers [2–3]. In the presence of AgSCF_3_, chloroiodane **2a** afforded an isolable and powerful electrophilic SCF_3_ source, which was used, for example, in α-ketone functionalizations among other reactions [2–3]. While at the time the proposed cyclic hypervalent iodine structure **5a** appeared reasonable in analogy to other well-established transfer reagents, it was unequivocally demonstrated to exist as the acyclic thioperoxide tautomer **5b** by Buchwald and co-workers in 2014 [4]. The structural reassignment was prompted by a series of remarkable, detailed inspections of ^1^H NMR spectra of precursors and congeners. A final structural corroboration came about by successfully encapsulating **5b**, an oil under ambient conditions, in a metal-organic framework (**5b**@MOF). This non-trivial protocol rendered it amenable to X-ray diffraction studies confirming the aforementioned structural reassignment. From a theoretical standpoint, acyclic isomer **5b** is predicted to be thermodynamically favored over the cyclic form **5a** by more than 10 kcal/mol by DFT calculations [5]. However, this type of computational analysis is in general still not decisive. For example, while Togni reagent **4a** is thermodynamically less favorable than its acyclic isomer **4b** by over 50 kcal/mol, a high kinetic barrier suppresses the [**a** → **b**] isomerization (Figure 1) [5–6].

With SCF_3_ reagent **5a**/**5b**, structure determination was notably challenging and solely provides a solid-state structural perspective. Thus, we wondered whether a correct structural assignment of reagent **5a**/**b** would have been feasible without having to resort to the preparation of crystalline congeners and/or the preparation of **5b**@MOF. Importantly, establishing a reliable way to differentiate cyclic (**a**) from acyclic (**b**) isomers in solution would facilitate future structure determination of similar iodine-based group-transfer reagents and provide greater mechanistic insight into reactivity of these reagents (Figure 1). Accordingly, we describe herein how ^17^O NMR spectroscopy in tandem with gauge-independent atomic orbital (GIAO) calculations may be a viable approach to establishing the predominant tautomer in solution.

## Results and Discussion

Arguably, the most common methods for structural elucidation of small organic molecules are one-dimensional ^1^H and ^13^C NMR spectroscopic techniques in combination with suitable two-dimensional experiments (COSY, HMBC, NOESY, etc.) [7]. However, in many cases (e.g., **5a** versus **5b**) these may only provide limited information, as neither nucleus is a primary constituent of the central iodine(III) (**a**, X–I–O) or iodine(I) (**b**, O–X) motif of interest. In stark contrast, changes in the oxygen ligand's environment should be readily traceable upon oxidation from alcohol **1** to chloroiodane **2a** (Figure 1, maroon), as well as during ensuing ligand substitutions, for example to fluoroiodane **3a**. In particular, whether oxygen is covalently bound to iodine or another element may heavily influence its shielding and thereby provide structural information by means of ^17^O NMR spectroscopy.

While natural abundance ^17^O NMR has been employed previously, including the analysis of hypervalent iodine compounds [8–10], this spectroscopic method has not yet found its entry into the organic chemist's standard NMR toolbox. This, in large part, may be attributed to the extremely low natural abundance of the ^17^O isotope (<0.04%) [11–12]. Consequently, the experiment requires high sample concentrations and relatively long experimental times, and ultimately fairly broad signals are observed. Yet, due to the large chemical shift range available (>1000 ppm), the technique may still prove diagnostic, especially when paired with calculated oxygen isotropic shift values. In order to substantiate this working hypothesis, five pairs of cyclic (**a**) vs acyclic (**b**) structural isomers **2**–**6** were investigated initially by DFT at the ωB97XD/aug-cc-pVDZ (aug-cc-pVDZ-PP basis set for iodine [6,13]) level of theory using Gaussian 09 [14–15]. The ωB97XD functional was chosen as a reasonably cost-effective way to include long-range dispersion [14].

Geometry optimizations of both cyclic and acyclic isomers were followed by calculation of oxygen isotropic shift values (δ_iso_) using the GIAO method (Table 1) [8–10]. Furthermore, these computed isotropic shift values (δ_iso_) were not referenced, for example to water, since they were directly correlated to experimentally determined ^17^O NMR shifts (vide infra). In addition, note that the calculations did not include treatment of spin-orbit-induced heavy-atom effects [16]. While undoubtedly important in the framework of classical bonding paradigms, they will only have a negligible effect on oxygen shifts derived for hypervalent iodine species. Specifically, spin-orbit effects heavily depend on and propagate through s-character rich bonds. However, within classical bonding theory the hypervalent bond about iodine comprises purely of p-orbitals (Rundle-Pimentel model) and most recently, this notion was corroborated for structure **4a** in a computational study [17]. Hence, effects on oxygen isotropic shifts will be minor at best and systematic and therefore, be accounted for by the abovementioned referencing to experimentally determined values.

We found that the calculated δ_iso_-values for the two isomers **2a**/**b** and **4a/b** differ by *∆*δ_iso_ ≈ 20; these differences are significantly larger for **3a**/**b** (*∆*δ_iso_ = −399.1), **5a**/**b** (*∆*δ_iso_ = 81.2) and **6a**/**b** (*∆*δ_iso_ = 52.7). Given that the larger the difference *∆*δ_iso_, the more likely a successful structural assignment based on ^17^O NMR spectroscopy becomes, this technique may indeed prove useful for the identification of the isomeric pairs **2**–**6**. Accordingly, spectral data on **1**, **2a**, **3a**, **4a**, **5** (assuming no assignment ), and **6** (unassigned) were acquired and further supplemented with values from some additional, structurally well-characterized hypervalent iodine compounds available in the literature (see Table 1 and Supporting Information File 1). Thus, a data set with a total of 11 entries was obtained.

**Table 1:** Compilation of δ_iso,_ δ_obs_ and δ_calc_ values.

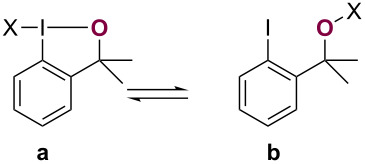					

entry	tautomer	δ_iso_^a^	δ_obs_^b^ [ppm]	δ_calc_^c^ [ppm]	|δ_calc_ – δ_obs_|

**1**	–	249.7	67	42	25
**2** (X = Cl)	**a**	192.7	116	116	0
	**b**	203.6	–	102	–
**3** (X = F)	**a**	236.2	59	60	1
	**b**	−162.9	–	575	–
**4** (X = CF_3_)	**a**	180.1	130	132	2
	**b**	203.8	–	101	–
**5** (X = SCF_3_)	**a**	173.6	–	140	–
	**b**	254.8	32	36	4
**6** (X = CN)	**a**	186.3	115	124	9
	**b**	239.0	–	56	–

^a^δ_iso_: computed isotropic shift value; ^b^δ_obs_: observed (experimental) chemical shift; ^c^δ_calc_: calculated (predicted) chemical shift.

**Figure 2:**
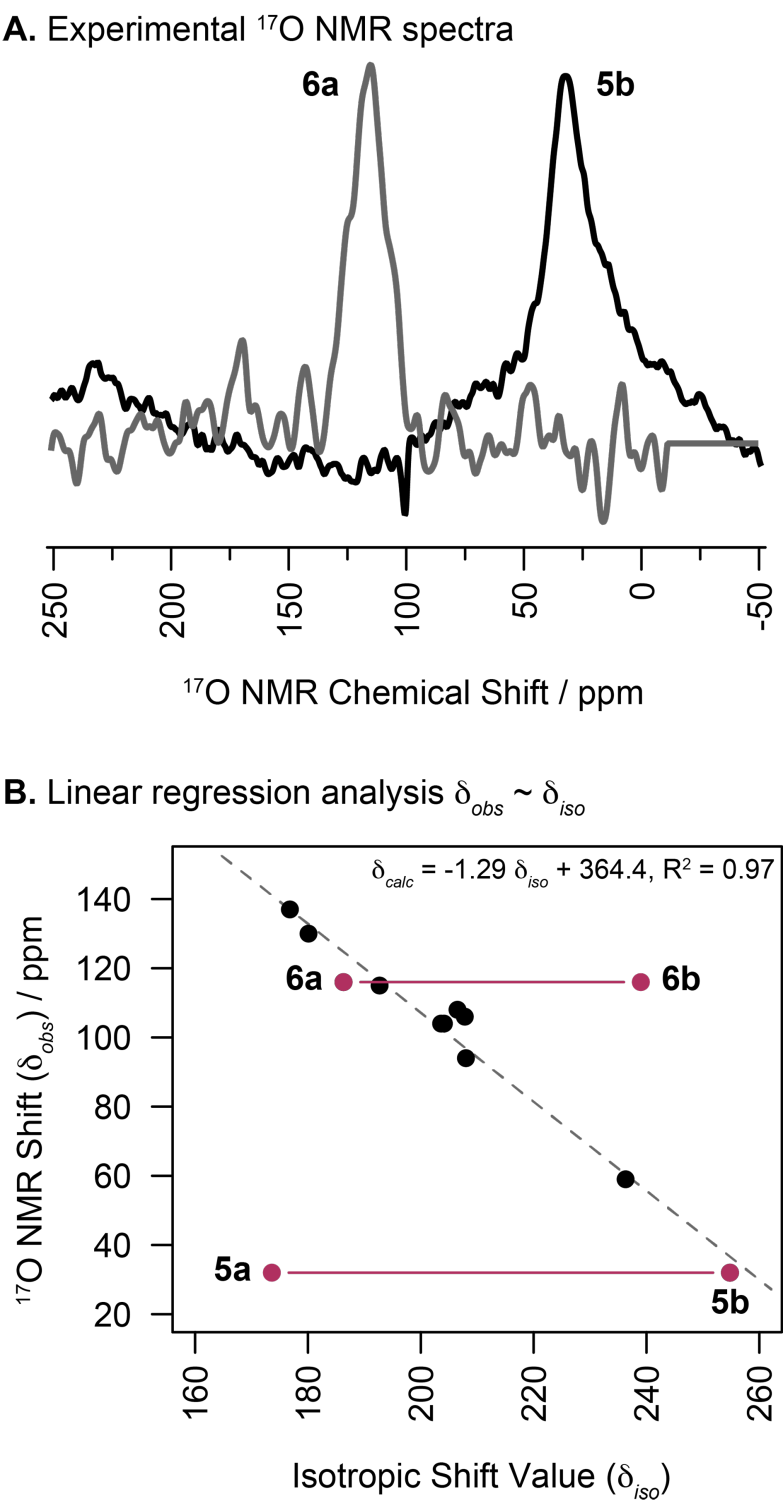
Assignment of acyclic (**b**) and cyclic (**a**) structures to **5** and **6**, respectively, based on computed isotropic shift values (δ_iso_) and experimental ^17^O NMR chemical shifts (δ_obs_).

To obtain experimental ^17^O NMR shifts, we used samples prepared in chloroform-*d* at a concentration of approximately 1.3 M. The obtained resonances typically featured a full-width at half maximum of around 1000–1500 Hz (Figure 2A). Therefore, the uncertainties of the determined ^17^O chemical shift values δ_obs_ are rated at a minimum of ±10 ppm, and thus, a reliable structural assignment should become feasible if predicted shift differences between the constitutional isomers **a** and **b** are greater. The observed ^17^O NMR chemical shifts ranged from 32 ppm (**5**) to 137 ppm (a C_2_F_5_-transfer reagent). Compounds **2a** and **4a** resonate at similar frequencies, with respective chemical shifts of 116 ppm and 130 ppm. For compound **5**, an approximately 100 ppm smaller chemical shift value was observed with δ_obs_ = 32 ppm, and for unassigned structure **6** we measured 115 ppm. It is noteworthy to indicate that under certain circumstances the absolute ^17^O NMR shift alone may be misleading in structure determination. For instance, the experimental value of 59 ppm for the known cyclic fluoroiodinane **3a** is closer to the observed values of acyclic **1** (67 ppm) and **5b** (32 ppm) than it is to cyclic **2a** and **4a**. However, assessment of the DFT-calculated isotropic shift values (δ_iso_) in tandem with experimental ^17^O NMR data (δ_obs_) lends credence to the aforementioned structural assignment. Specifically, for the "unassigned" compounds mentioned above, the best R^2^-value for a linear relationship δ_obs_ ~ δ_iso_ is obtained when **5** and **6** are assigned as **5b** and **6a**, where the additional known compounds serve as calibration (Figure 2B) [18]. Based on the thus derived equation, ^17^O NMR chemical shifts δ_calc_ can be predicted for both isomers. A notable exception is the free alcohol **1**, which is not part of the linear relationship and consequently displays a large residual value (Table 1). Conceivably, this may be due to intermolecular hydrogen bonding with the solvent or other alcohol molecules in the concentrated solution. In fact, including a methanol solvent molecule as a hydrogen-bond donor in the DFT calculation will shift the δ_calc_ in the right direction for **1** (i.e., to δ_calc_ = 47 ppm based on δ_iso_ = 245.8, although |δ_calc_ – δ_obs_| is still 20 ppm).

For the pair **5a**/**b** a difference ∆δ_calc_ of 104 ppm is obtained and a value of 68 ppm results for **6a**/**b** (Figure 2). Both figures are significantly larger than the ^17^O chemical shift's lower-bound uncertainty estimate of ±10 ppm. While **5a**/**b** indeed has been shown to exist as the thioperoxide **5b** (vide infra), a crystallographic study on **6a/b** is required to corroborate our prediction as **6a**.

**Figure 3:**
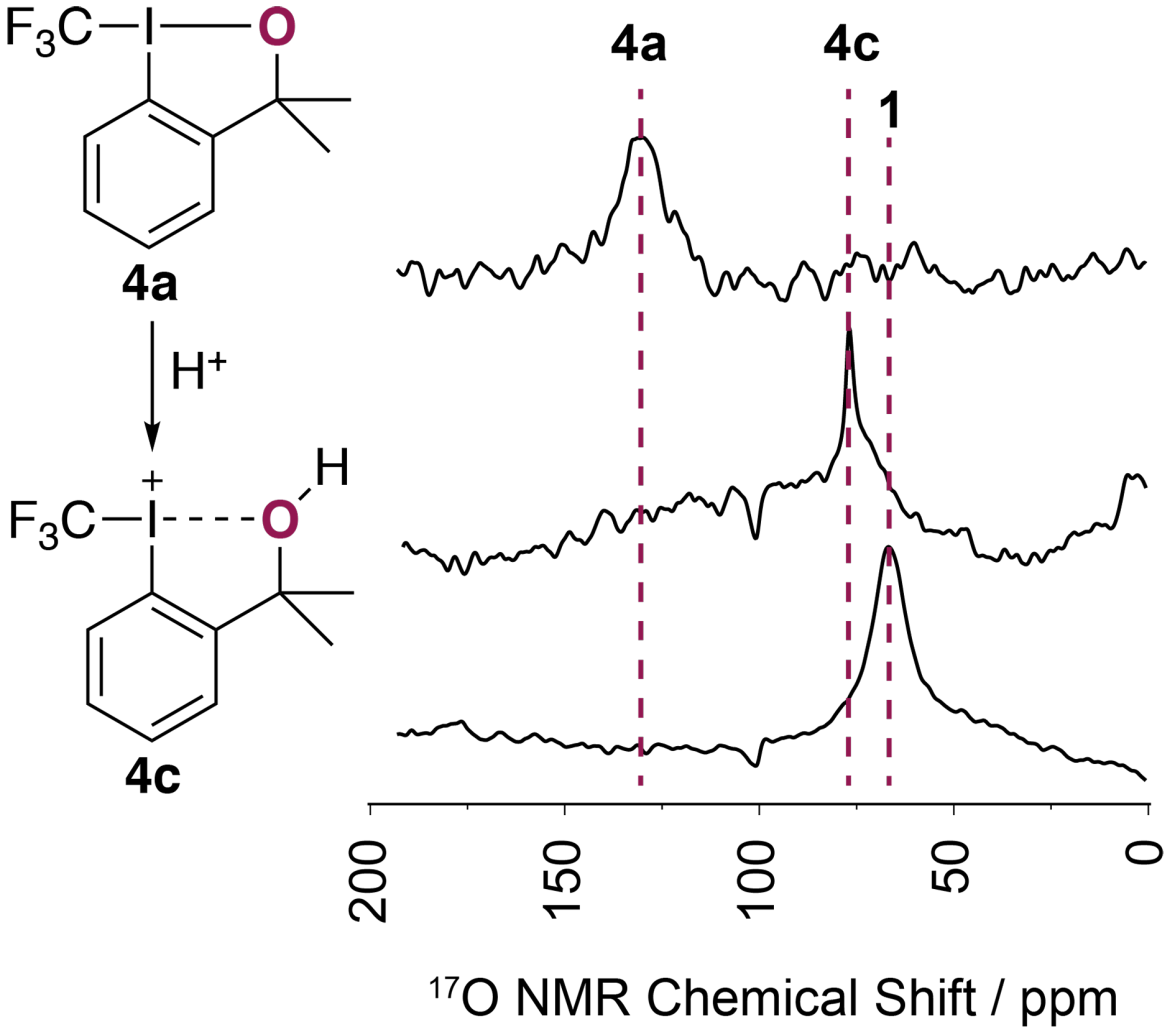
Protonation of **4a** with trifluoroacetic acid (5 equiv) affords **4c**, followed by ^17^O NMR spectroscopy.

To further gauge the utility of this approach, the activation of Togni reagent **4a** was studied, in particular its protonation with a strong acid [1]. This brings about a significant elongation of the I–O bond from 2.203(5) Å in **4a** to 2.4991(13) Å in the fully protonated form **4c** [1,19]. Most recently, Toste and co-workers studied this activation strategy too and demonstrated that in the presence of an equivalent of gaseous HCl compound **4a** afforded an isolable iodonium-type structure [20]. Although this activation can be conveniently followed by ^19^F NMR spectroscopy with **4a** resonating at −40.1 ppm and the fully protonated “iodonium” congener **4c** at −20 ppm [1], this technique provides no indication on how to best represent **4c** in solution. Does the compound resemble the molecular structure obtained in the solid state with oxygen still coordinated to iodine or would a free alcohol be a more accurate representation? In order to generate **4c**, reagent **4a** was treated with five equivalents of trifluoroacetic acid (TFA) and then subjected to spectroscopic analysis. A ^19^F NMR chemical shift of −23.2 ppm was obtained, thereby confirming the presence of **4c**. However, under these strongly acidic and activating conditions, the compound is unstable over a prolonged period of time (12 h). During the acquisition of the ^17^O NMR data, approximately 36% of **4c** had decomposed to the corresponding α-methylstyrene derivative as indicated by ^1^H NMR spectroscopy (see Supporting Information File 1). As this byproduct is ^17^O NMR silent, the spectral data acquisition was unhampered and a chemical shift δ_obs_ = 77 ppm was measured. This value is larger than the chemical shift obtained for the free alcohol **1** (67 ppm) and at the same time, also significantly smaller than the value obtained for the native reagent **4a** (130 ppm). Structure **4c** was computed in the gas phase in absence of a counter anion and geometry optimization furnished a minimum reminiscent of the pictographic representation of **4c** with an intact but significantly elongated I–O bond of 2.55 Å (Figure 3) and qualitatively, the NMR data are in support of this notion. From a quantitative point of view, the data points (δ_iso_, δ_obs_) for **1**, **4b**, and **4c** afforded a perfect linear correlation with R^2^ = 1, thus lending further credence to the representation of the protonated form **4a** in solution as **4c** (see Supporting Information File 1).

## Conclusion

In summary, the present study demonstrates that ^17^O NMR spectroscopy at natural abundance coupled with DFT-calculated isotropic shift values can be used to gain insight into the solution-phase tautomerism observed in iodine-based group-transfer reagents. In particular, we confirm that Shen’s revised, electrophilic SCF_3_-transfer reagent adopts an "acyclic" thioperoxide tautomeric form in solution whereas an electrophilic cyanide source prefers the "cyclic" iodane. Since ^17^O NMR experiments are easily implemented on contemporary spectrometers, this method may provide the most convenient spectroscopic handle to re-evaluate known structures, facilitate further mechanistic studies, and provide a complimentary approach to solid-state structural analysis.

## Supporting Information

File 1 ^17^O NMR spectra and calculated molecular geometries.
