# Supplementary material for: Determining the predominant tautomeric structure of iodine-based group-transfer reagents by 17O NMR spectroscopy
Source: Beilstein J Org Chem. 2018 Aug 30;14:2289–94. doi: 10.3762/bjoc.14.203 (PMC6122331; doi:10.3762/bjoc.14.203)
Supplement: File 1 — 17O NMR spectra and calculated molecular geometries. [file Beilstein_J_Org_Chem-14-2289-s001.pdf]

Supporting Information

for

Determining the predominant tautomeric structure of iodine-based group-transfer reagents by ^{17}O NMR spectroscopy

Nico Santschi*, Cody Ross Pitts, Benson J. Jelier and René Verel

Address: Eidgenössische Technische Hochschule (ETH) Zürich, Department of Chemistry and Applied Biosciences, Vladimir-Prelog-Weg 1/2, 8093 Zürich, Switzerland

Email: Nico Santschi* - snico@inorg.chem.ethz.ch

*Corresponding author

^{17}O NMR spectra and calculated molecular geometries

Table of contents

General information ^{17}O NMR S2
^{17}O NMR of 2-(2-iodophenyl)propan-2-ol (**1**) S3
^{17}O NMR of 1-chloro-3,3-dimethyl-1,2-benziodoxole (**2a**) S3
^{17}O NMR of 1-fluoro-3,3-dimethyl-1,2-benziodoxole (**3a**) S4
^{17}O NMR of 1-trifluoromethyl-3,3-dimethyl-1,2-benziodoxole (**4a**) S4
^{17}O NMR of ((2-(2-iodophenyl)propan-2-yl)oxy)(trifluoromethyl)sulfane (**5b**) S5
^{17}O NMR of 1-cyano-3,3-dimethyl-1,2 -benziodoxole (**6a**) S5
^{17}O NMR of 1-chloro-3-ethyl-3-methyl-1,2-benziodoxole S6
^{17}O NMR of 1-chloro-3-methyl-3-propyl-1,2-benziodoxole S6
^{17}O NMR of 1-chloro-3-methyl-3-isobutyl-1,2-benziodoxole S7
^{17}O NMR of 1-chloro-3-methyl-3-isopropyl-1,2-benziodoxole S7
^{17}O NMR of 1-trifluoro-3-methyl-3-isopropyl-1,2-benziodoxole S8
^{17}O NMR of 1-(pentafluoroethyl)-3-methyl-3-isopropyl-1,2-benziodoxole S8
^{17}O NMR of **4a** + TFA (5 equiv) → **4c** S9
^{19}F NMR of **4a** + TFA (5 equiv) → **4c** (t = 0) S9
^{1}H NMR of **4a** + TFA (5 equiv) → **4c** (t = 0) S10
^{19}F NMR of **4a** + TFA (5 equiv) → **4c** (t = 12 h) S10
^{1}H NMR of **4a** + TFA (5 equiv) → **4c** (t = 12 h) S11
Correlation $\delta_{iso} \sim \delta_{obs}$ for **4a** + TFA(5 equiv) → **4c** (t = 12 h) S11
Computational methods S11
Coordinates of calculated structures S12

General Information ^{17}O NMR

^{17}O NMR experiments were performed at ambient temperature on an Avance III spectrometer (Bruker Biospin, Fällanden, Switzerland) equipped with a 5 mm PABBO probehead and at a magnetic field of 9.4 T (54.2 MHz Larmor Frequency for ^{17}O). A simple one-pulse excitation acquire pulse sequence was used with a pulse length of 9 μs and a recycle delay of 0 s. A total of between 120k and 770k scans were co-added for each of the experiments leading to a total acquisition time per experiment between 2.5 and 17 hours. A spectral width of 500 ppm (27.1 kHz) was acquired with 2048 points.

All spectral data were processed with MestReNova v11.0.2-18153. The FID's were zero filled to 2048k and apodized with an exponential function of 128 or 256 Hz prior to Fourier Transformation and phasing. Due to severe ringing and the baseline distortions this causes, multipoint baseline corrections (cubic splines) were applied. Chemical shifts (ppm) and widths (Hz) were derived by line fitting using the Generalized Lorentzian shape type.

Example of compound **4a** without baseline correction:

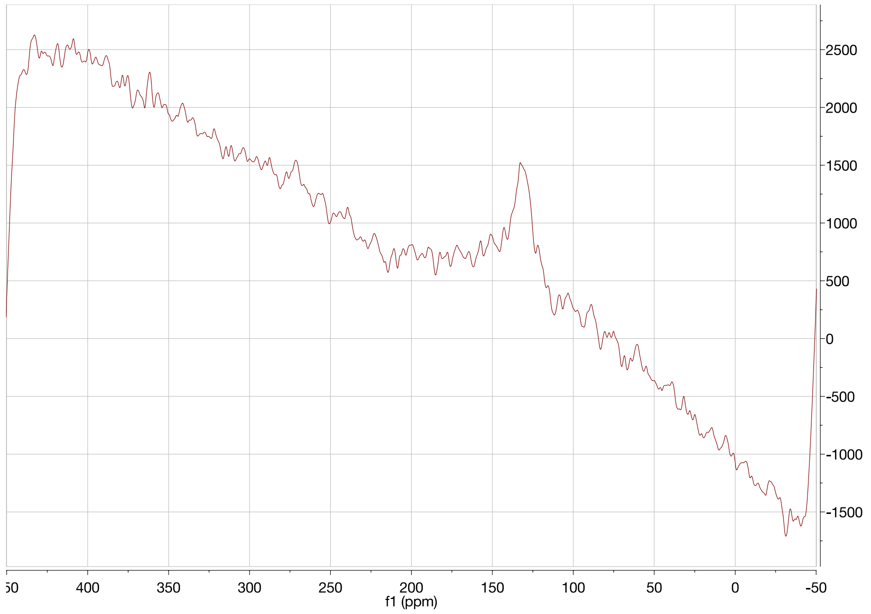

^{17}O NMR of 2-(2-iodophenyl)propan-2-ol (1)

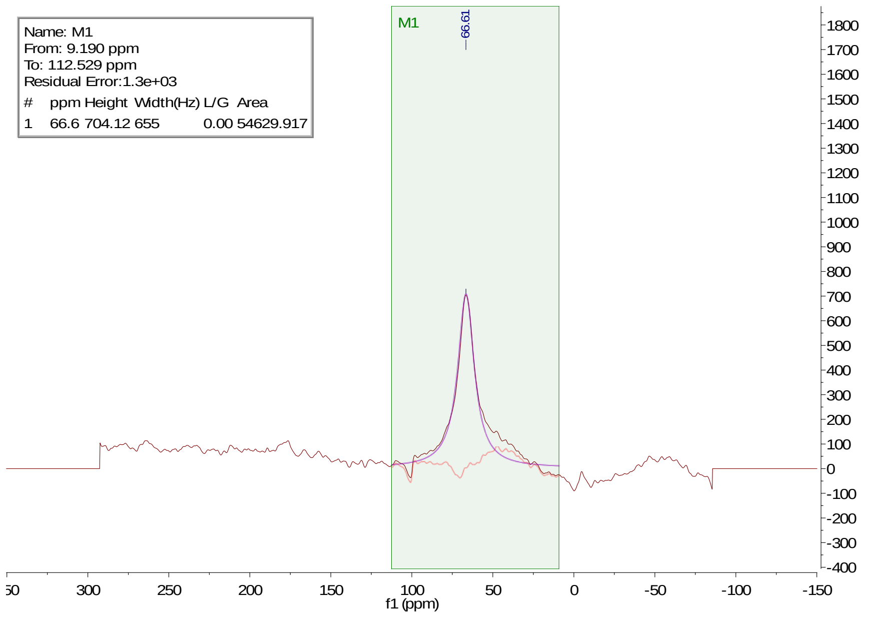

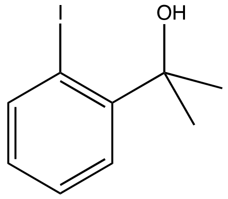

^{17}O NMR of 1-chloro-3,3-dimethyl-1,2-benziodoxole (2a)

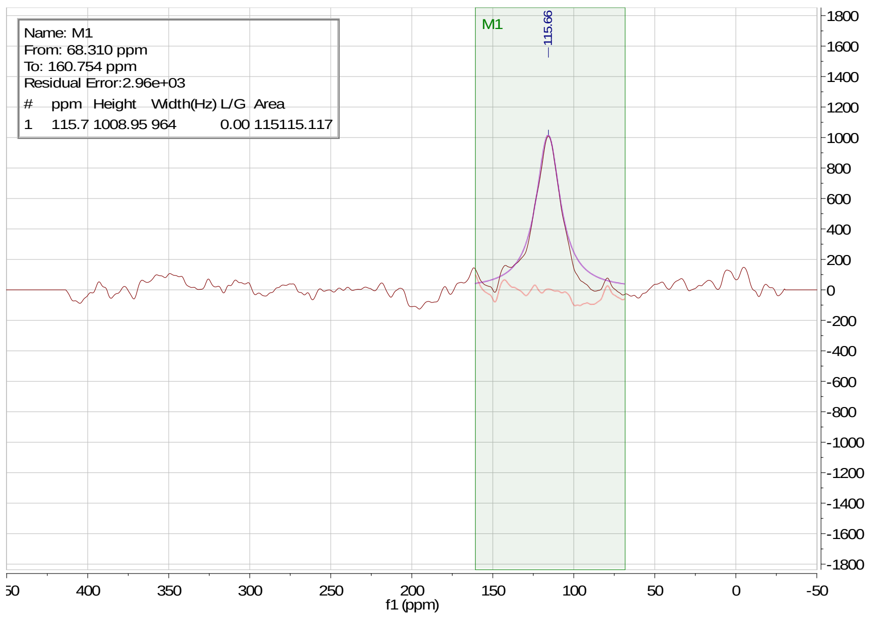

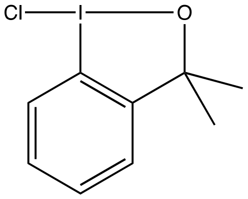

^{17}O NMR of 1-fluoro-3,3-dimethyl-1,2-benziodoxole (3a)

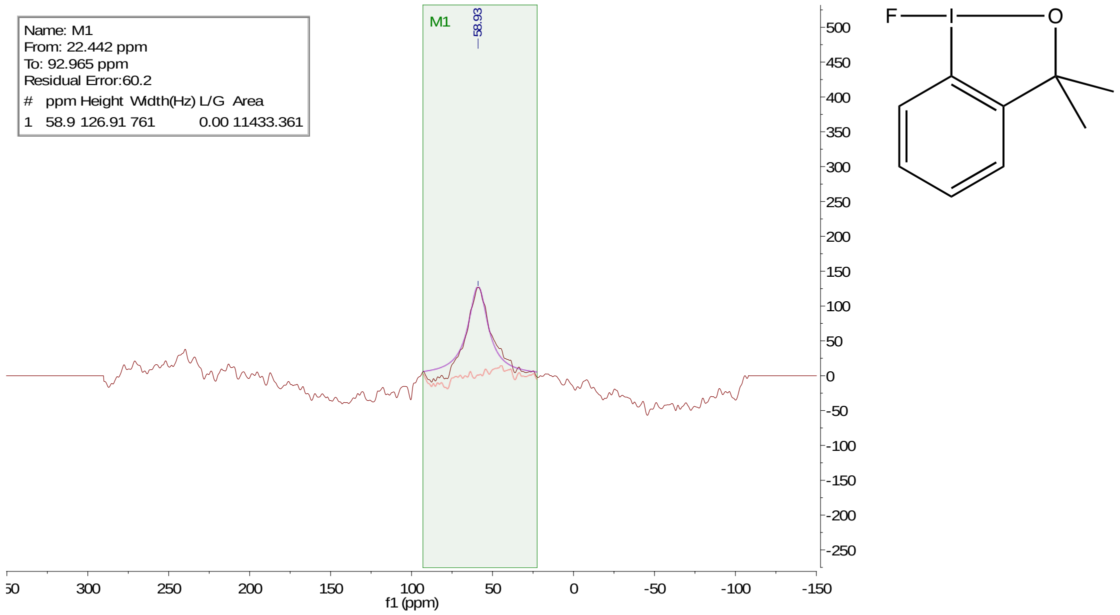

^{17}O NMR of 1-trifluoromethyl-3,3-dimethyl-1,2-benziodoxole (4a)

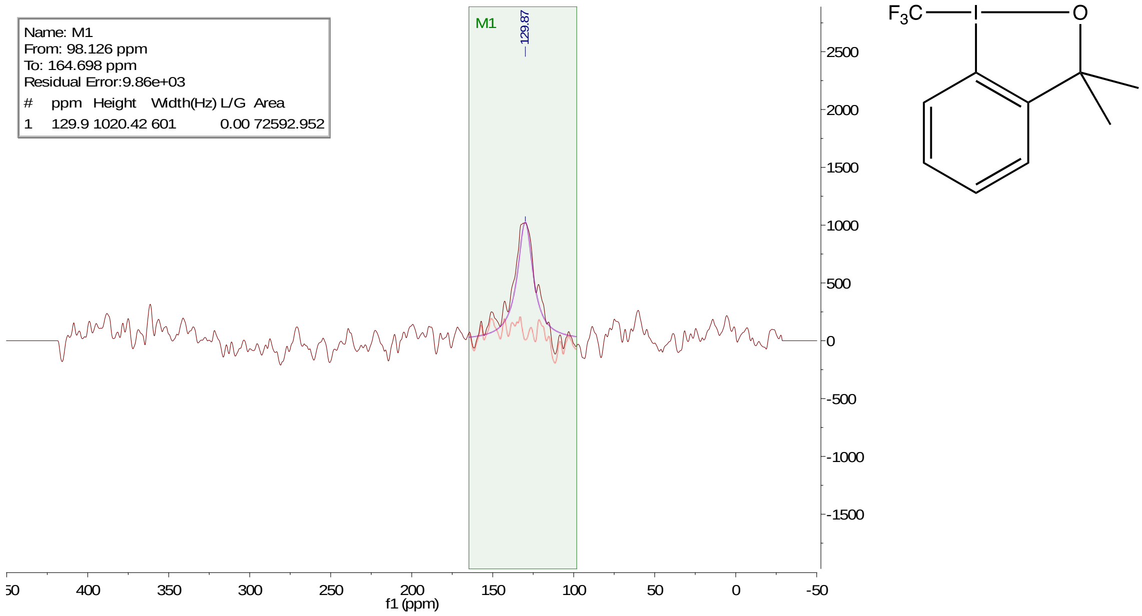

^{17}O NMR of ((2-(2-iodophenyl)propan-2-yl)oxy)(trifluoromethyl)sulfane (5b)

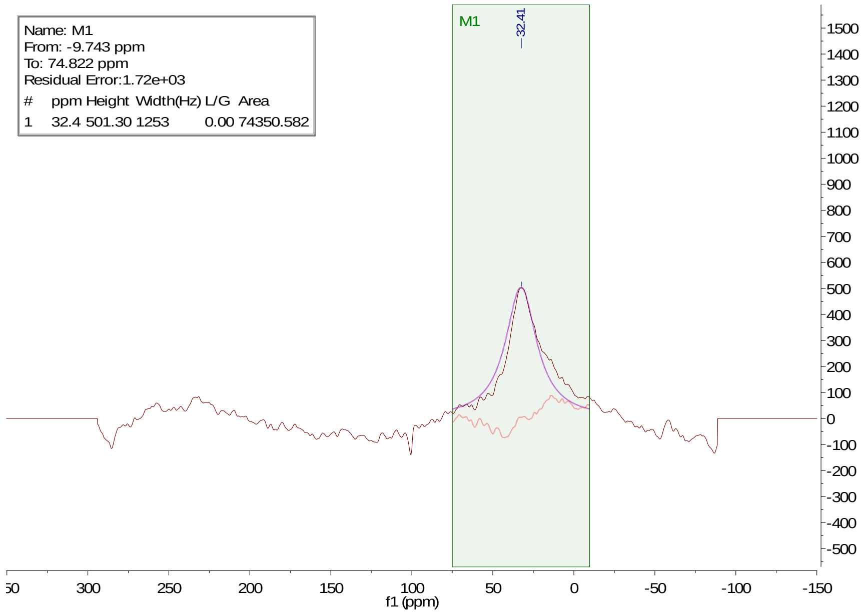

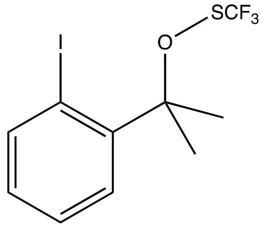

^{17}O NMR of 1-cyano-3,3-dimethyl-1,2 -benziodoxole (6a)

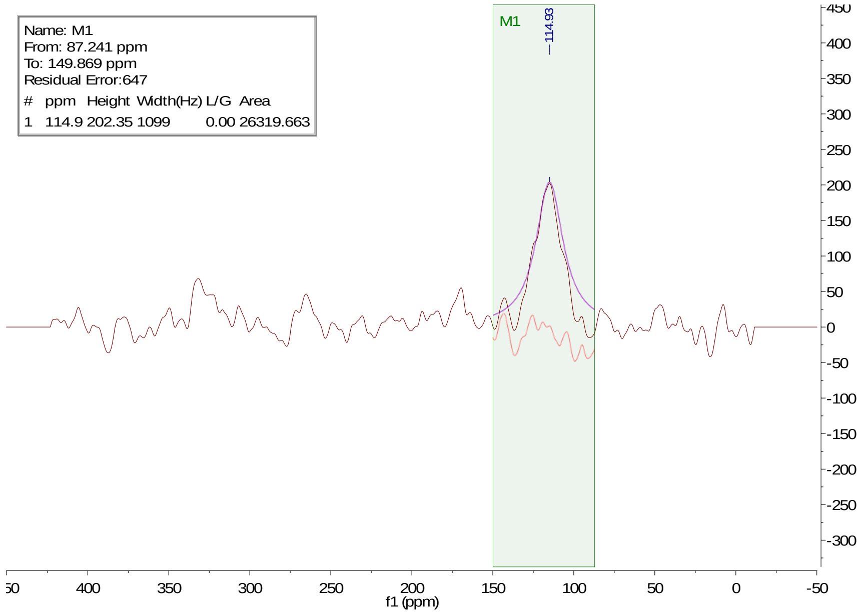

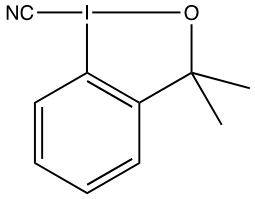

^{17}O NMR of 1-chloro-3-ethyl-3-methyl-1,2-benziodoxole

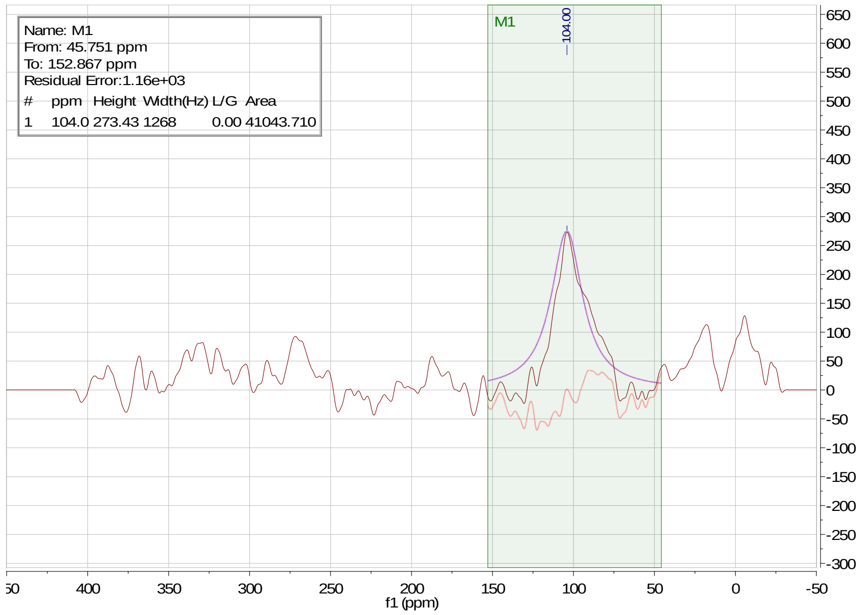

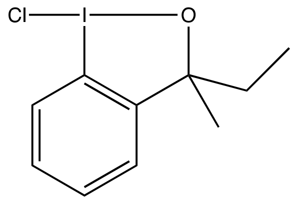

^{17}O NMR of 1-chloro-3-methyl-3-propyl-1,2-benziodoxole

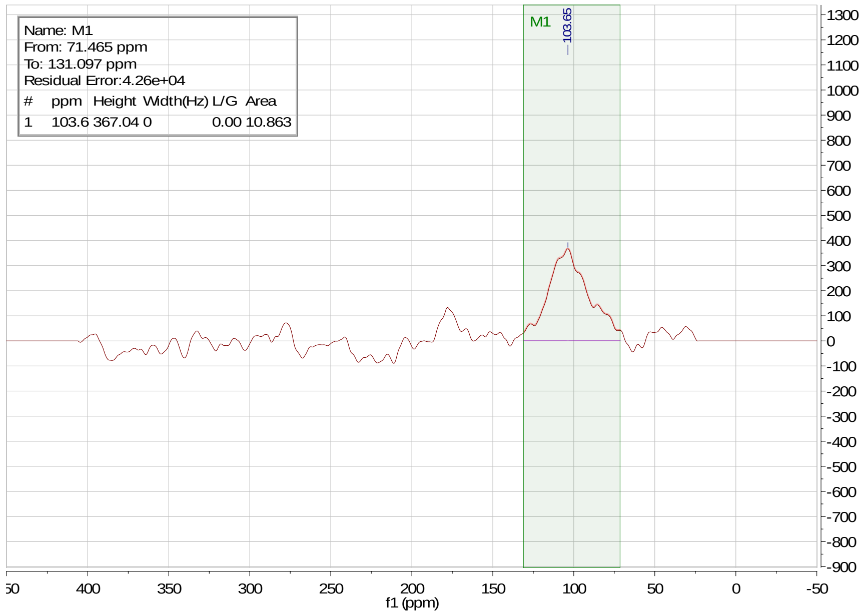

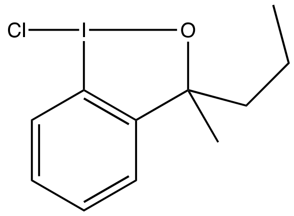

^{17}O NMR of 1-chloro-3-methyl-3-isobutyl-1,2-benziodoxole

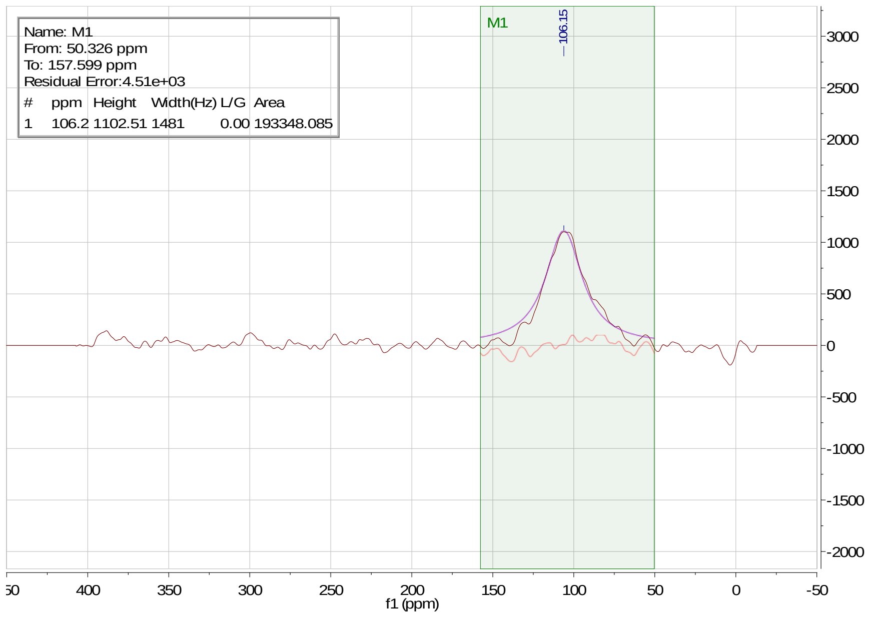

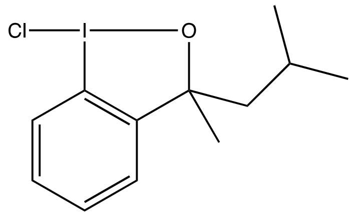

^{17}O NMR of 1-chloro-3-methyl-3-isopropyl-1,2-benziodoxole

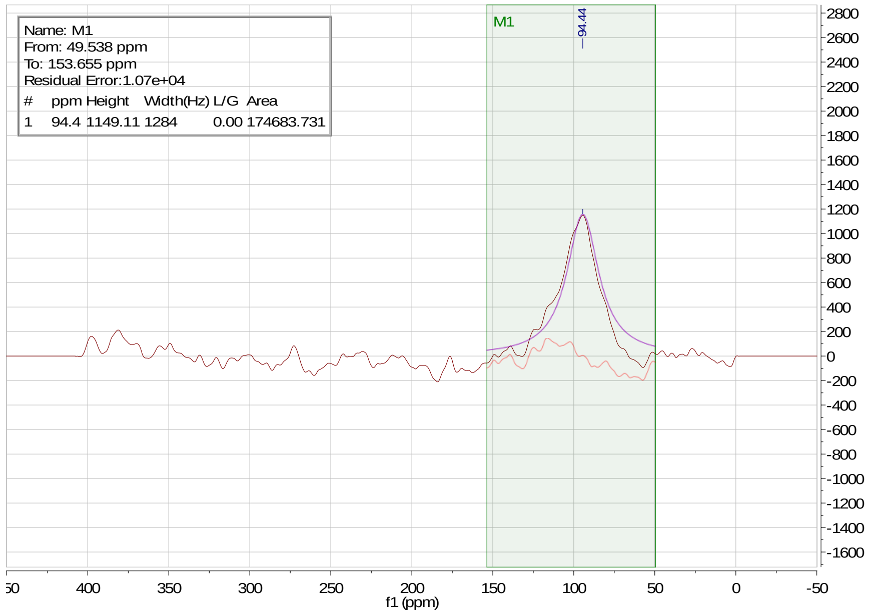

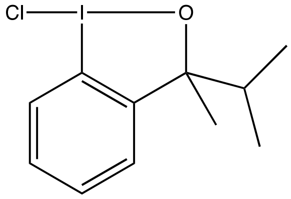

^{17}O NMR of 1-trifluoro-3-methyl-3-isopropyl-1,2-benziodoxole

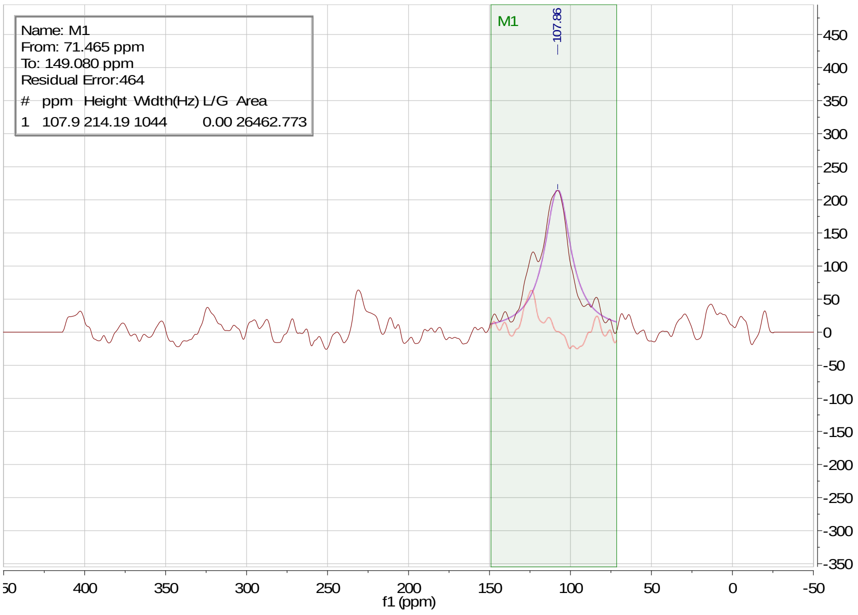

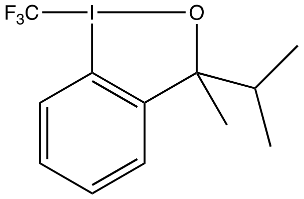

^{17}O NMR of 1-(pentafluoroethyl)-3-methyl-3-isopropyl-1,2-benziodoxole

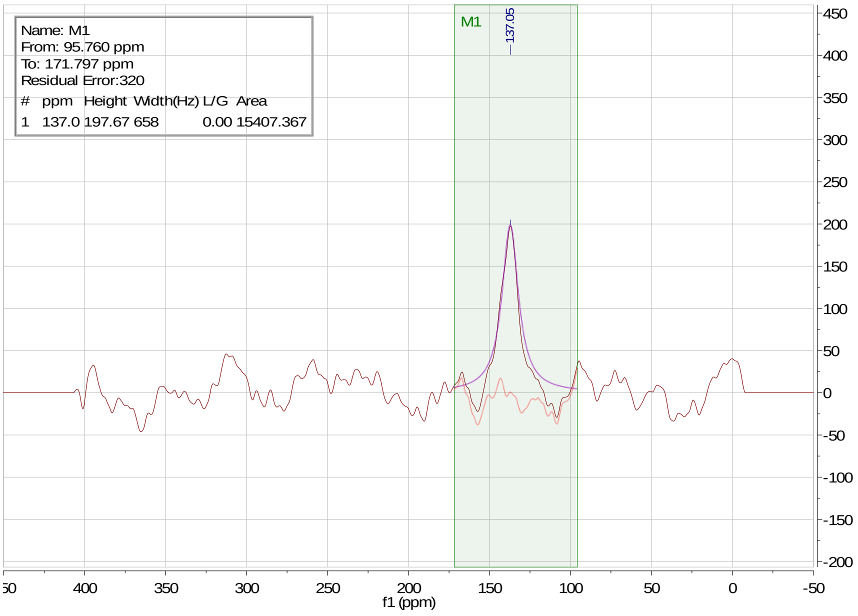

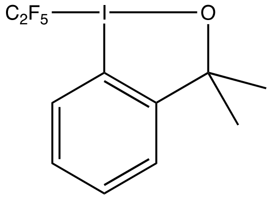

^{17}O NMR of 4a + TFA (5 equiv) ⟶ 4c

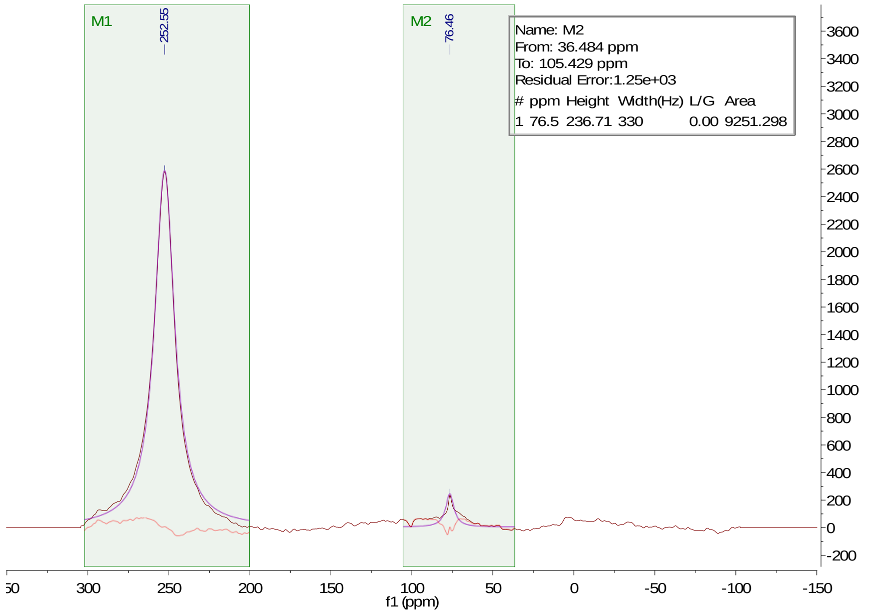

^{19}F NMR of 4a + TFA (5 equiv) ⟶ 4c (t = 0)

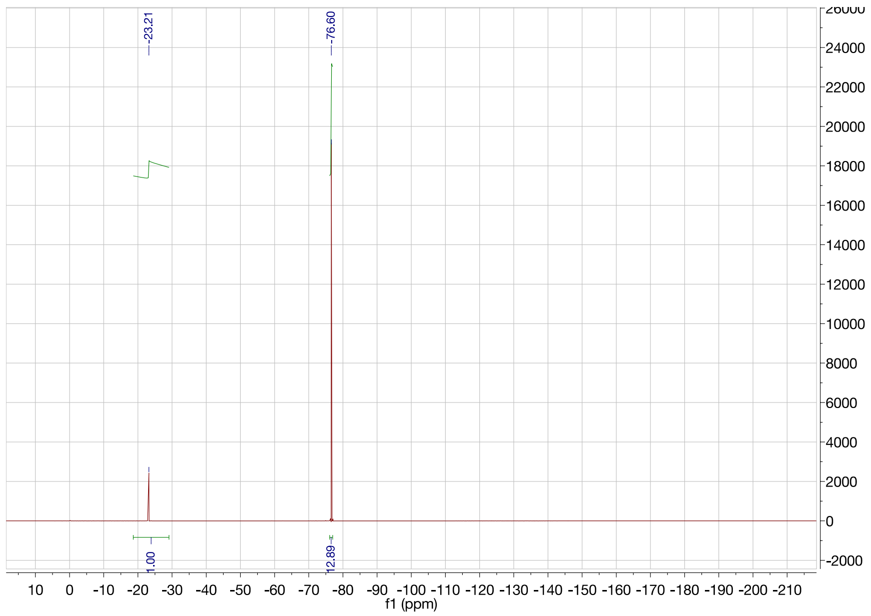

^{1}H NMR of 4a + TFA (5 equiv) ⟶ 4c (t = 0)

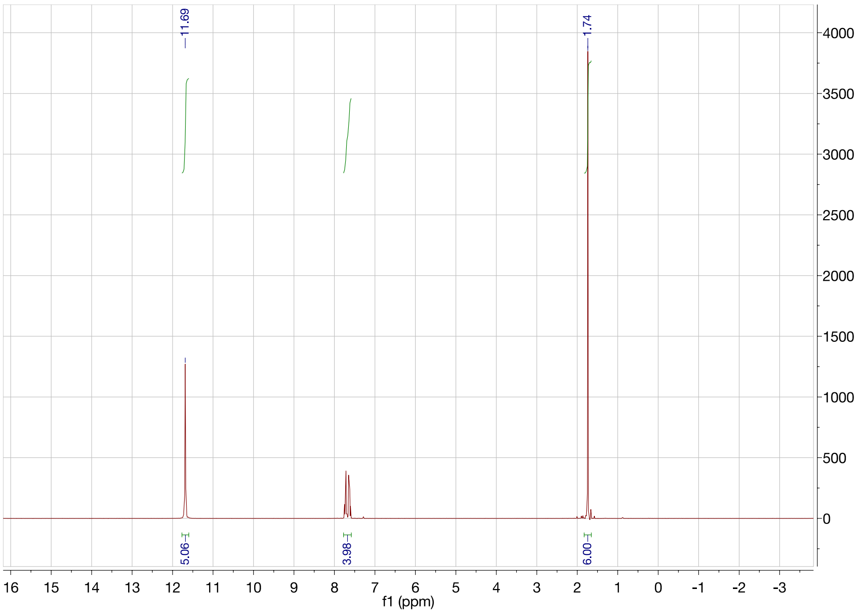

^{19}F NMR of 4a + TFA (5 equiv) ⟶ 4c (t = 12 h)

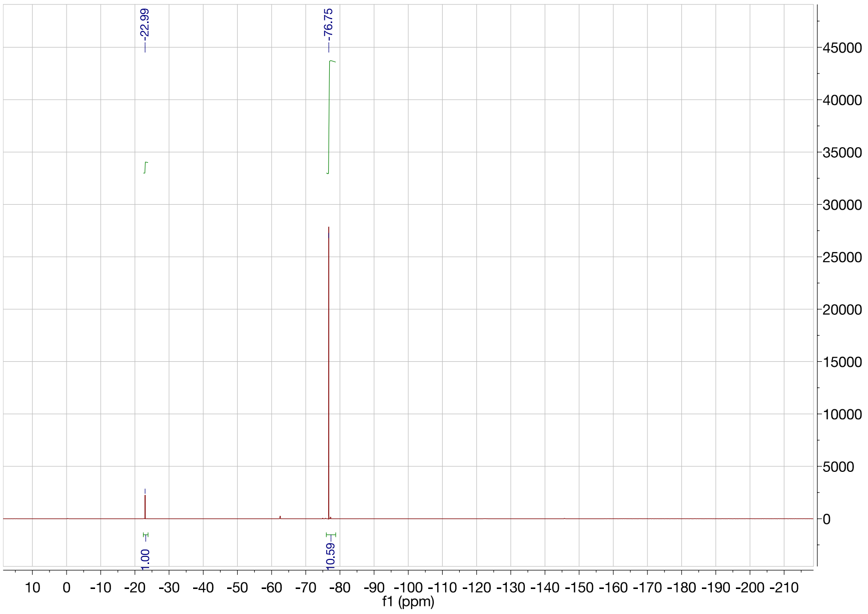

^{1}H NMR of 4a + TFA (5 equiv) ⟶ 4c (t = 12 h)

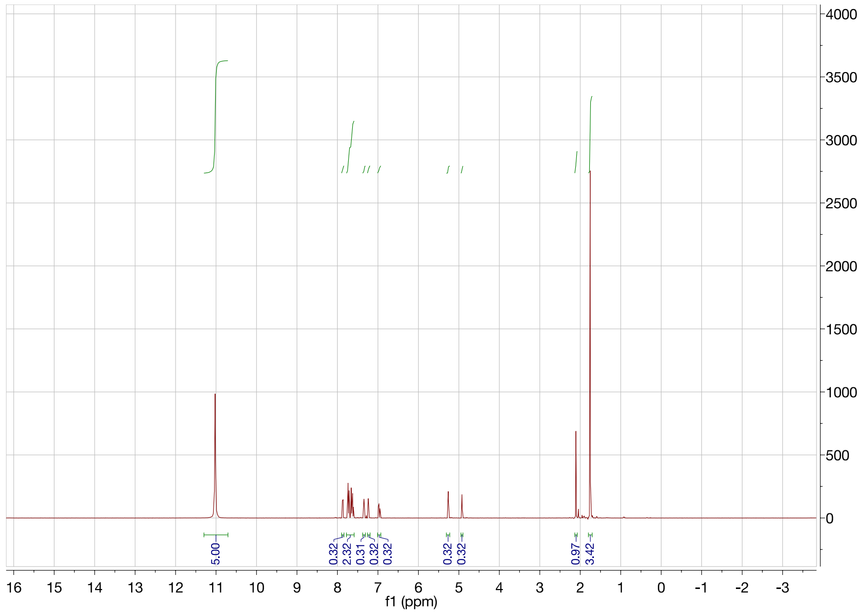

Correlation δ_{iso} ~ δ_{obs} for 4a + TFA (5 equiv) ⟶ 4c (t = 12 h)

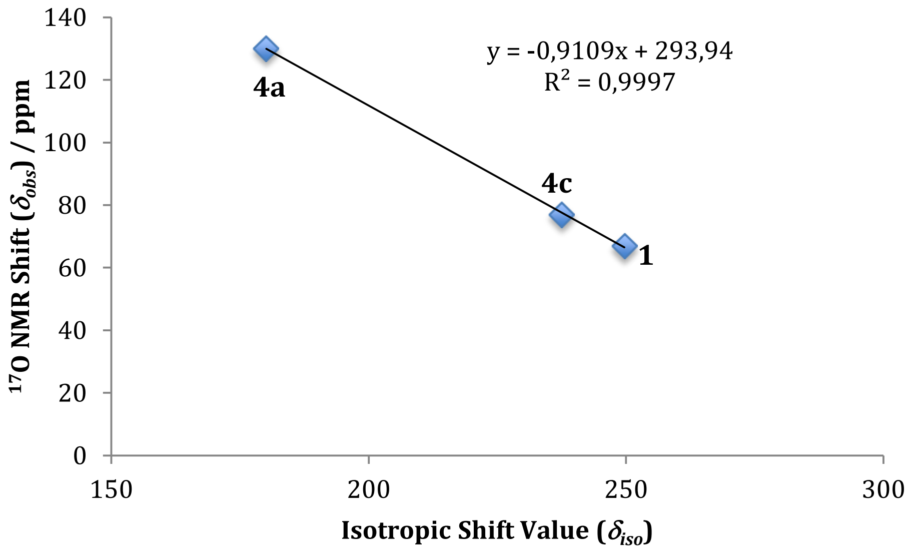

Computational methods

The Gaussian '09 package was used for all geometry optimizations. The calculated ^{17}O NMR shifts (δ_{calc}) were determined by fitting the isotropic values (δ_{iso}) from the GIAO calculations to the empirical equation $\delta_{calc} = -1.29(\delta_{iso}) + 364.4$. The equation was calibrated for this study at ωB97XD/aug-cc-pVDZ (using an aug-cc-pVDZ-PP basis set for iodine) by linear regression analysis between the calculated δ_{iso} values and experimental ^{17}O chemical shift (δ) values of iodine-based group transfer reagents with structural assignments confirmed by X-ray crystallography.

Coordinates of calculated structures

Compound 1

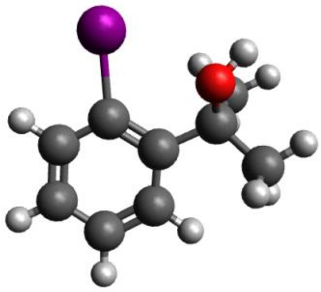

\# nmr=giao wb97xd/genecp geom=connectivity

Center Number	Atomic Number	Atomic Type	Coordinates (Angstroms) X	Y	Z
1	6	0	-2.425650	-2.284734	0.028716
2	6	0	-2.446857	-0.893636	0.000084
3	6	0	-1.280119	-0.116706	-0.027651
4	6	0	-0.059411	-0.818098	-0.018519
5	6	0	-0.028483	-2.215023	0.003284
6	6	0	-1.207981	-2.952820	0.025769
7	1	0	-3.363230	-2.839445	0.046904
8	1	0	-3.414751	-0.399628	-0.005480
9	1	0	0.929208	-2.732325	0.004711
10	1	0	-1.165146	-4.041575	0.042144
11	6	0	-1.396502	1.415535	-0.020299
12	6	0	-2.804179	1.915216	-0.367645
13	1	0	-3.541648	1.674061	0.408710
14	1	0	-2.763488	3.008296	-0.458069
15	1	0	-3.138013	1.507190	-1.329090
16	53	0	1.876374	0.067560	-0.010976
17	6	0	-1.000318	1.959650	1.359588
18	1	0	0.021453	1.673441	1.629109
19	1	0	-1.071945	3.057649	1.364801
20	1	0	-1.685121	1.573187	2.124738
21	8	0	-0.516208	1.888205	-1.041268
22	1	0	-0.228482	2.776538	-0.816539

Compound 1 + MeOH

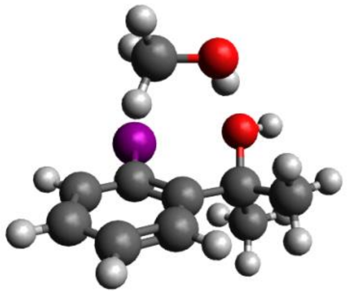

----
nmr=giao wb97xd/genecp geom=connectivity

Center Number	Atomic Number	Atomic Type	Coordinates (Angstroms) X	Y	Z
1	6	0	-2.090427	-2.170358	1.219500
2	6	0	-2.111478	-0.785027	1.096488
3	6	0	-0.989049	-0.041703	0.706111
4	6	0	0.188624	-0.768422	0.447456
5	6	0	0.215626	-2.161228	0.560773
6	6	0	-0.919922	-2.866536	0.944964
7	1	0	-2.993672	-2.699741	1.519967
8	1	0	-3.046349	-0.271038	1.300810
9	1	0	1.136676	-2.700047	0.346809
10	1	0	-0.880816	-3.952242	1.027191
11	6	0	-1.103843	1.485928	0.610689
12	6	0	-2.553211	1.981721	0.583194
13	1	0	-3.062182	1.829020	1.542883
14	1	0	-2.544431	3.062875	0.390138
15	1	0	-3.118465	1.493962	-0.219773
16	53	0	2.059193	0.063313	-0.133284
17	6	0	-0.355737	2.152316	1.771440
18	1	0	0.700987	1.867461	1.788078
19	1	0	-0.423430	3.246633	1.683347
20	1	0	-0.814099	1.859637	2.724274
21	8	0	-0.507916	1.836797	-0.648741
22	1	0	-0.407139	2.791505	-0.692744
23	8	0	-2.025803	0.390966	-2.607848
24	1	0	-1.454268	0.901430	-2.017625
25	6	0	-1.554949	-0.940786	-2.648940
26	1	0	-1.774202	-1.485870	-1.715676
27	1	0	-2.070174	-1.446327	-3.474893
28	1	0	-0.469732	-0.990374	-2.836046

Compound 2a

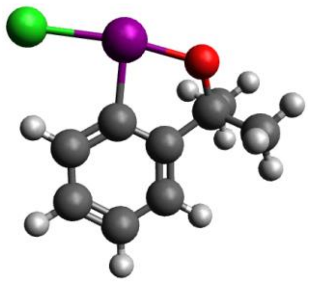

---

nmr=giao wb97xd/genecp geom=connectivity

Center Number	Atomic Number	Atomic Type	Coordinates (Angstroms) X	Y	Z
1	6	0	2.291784	2.682870	-0.006830
2	6	0	2.584021	1.320304	0.018979
3	6	0	1.558599	0.372160	-0.016155
4	6	0	0.261255	0.860914	-0.068845
5	6	0	-0.076874	2.202779	-0.086375
6	6	0	0.969857	3.124636	-0.059743
7	1	0	3.104345	3.408389	0.015702
8	1	0	3.621490	0.991434	0.068944
9	1	0	-1.118024	2.517658	-0.116999
10	1	0	0.746410	4.190553	-0.081274
11	6	0	1.773362	-1.139699	0.061480
12	6	0	2.925585	-1.595638	-0.833579
13	1	0	3.887112	-1.205995	-0.474099
14	1	0	2.974268	-2.691043	-0.822411
15	1	0	2.763788	-1.259977	-1.864274
16	8	0	0.607267	-1.779837	-0.435505
17	53	0	-1.140922	-0.735515	-0.106305
18	6	0	2.020987	-1.537247	1.523566
19	1	0	1.174669	-1.241199	2.157842
20	1	0	2.144873	-2.625092	1.591180
21	1	0	2.924626	-1.049790	1.912872
22	17	0	-3.086133	0.849387	0.207983

Compound 2b

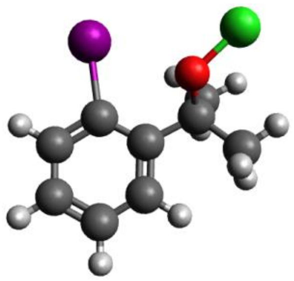

----
nmr=giao wb97xd/genecp geom=connectivity

Center Number	Atomic Number	Atomic Type	Coordinates (Angstroms) X	Y	Z
1	6	0	-2.209065	2.833447	-0.229817
2	6	0	-0.846356	2.614892	-0.056695
3	6	0	-0.293014	1.330582	0.043169
4	6	0	-1.188328	0.245525	-0.045708
5	6	0	-2.557213	0.459967	-0.227047
6	6	0	-3.072485	1.748897	-0.317985
7	1	0	-2.589171	3.851876	-0.300933
8	1	0	-0.194832	3.482290	0.002479
9	1	0	-3.229654	-0.392935	-0.296913
10	1	0	-4.142887	1.895467	-0.458928
11	6	0	1.218321	1.193718	0.294717
12	6	0	2.001182	2.484634	0.061243
13	1	0	1.768577	3.237424	0.824048
14	1	0	3.071844	2.262948	0.136739
15	1	0	1.802198	2.896027	-0.934972
16	53	0	-0.661720	-1.812329	0.066019
17	6	0	1.475253	0.683100	1.715420
18	1	0	1.011293	-0.291079	1.893255
19	1	0	2.551016	0.601931	1.907866
20	1	0	1.051515	1.406525	2.423120
21	8	0	1.593677	0.246417	-0.740968
22	17	0	3.179532	-0.378645	-0.599460

Compound 3a

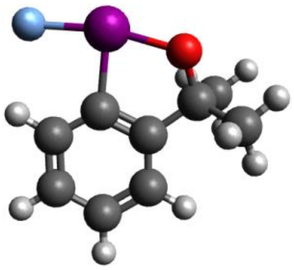

---

nmr=giao wb97xd/genecp geom=connectivity

Center Number	Atomic Number	Atomic Type	Coordinates (Angstroms) X	Y	Z
1	6	0	2.753243	2.049362	-0.010348
2	6	0	2.659626	0.658294	-0.013025
3	6	0	1.410574	0.032864	-0.032064
4	6	0	0.298932	0.860532	-0.043192
5	6	0	0.342329	2.245474	-0.029478
6	6	0	1.603658	2.840913	-0.017293
7	1	0	3.734898	2.522144	0.000424
8	1	0	3.567801	0.056276	0.002746
9	1	0	-0.576687	2.827227	-0.025361
10	1	0	1.685648	3.927280	-0.013495
11	6	0	1.192363	-1.480604	0.025194
12	6	0	2.120977	-2.220660	-0.937527
13	1	0	3.172492	-2.114961	-0.639926
14	1	0	1.867538	-3.287655	-0.930744
15	1	0	1.996076	-1.834787	-1.955870
16	8	0	-0.132377	-1.762270	-0.401638
17	53	0	-1.488905	-0.254471	-0.056573
18	6	0	1.396049	-1.961557	1.469058
19	1	0	0.702364	-1.450411	2.149789
20	1	0	1.209090	-3.041043	1.524891
21	1	0	2.420462	-1.757789	1.808459
22	9	0	-2.497248	1.510124	0.202514

Compound 3b

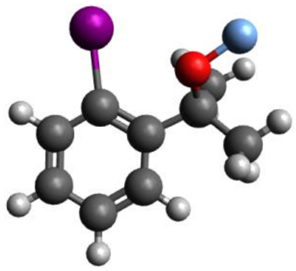

---

nmr=giao wb97xd/genecp geom=connectivity

Center Number	Atomic Number	Atomic Type	Coordinates (Angstroms) X	Y	Z
1	6	0	1.776780	2.951222	-0.099726
2	6	0	2.112410	1.604355	-0.017199
3	6	0	1.149071	0.586380	0.027876
4	6	0	-0.202646	0.985330	-0.022070
5	6	0	-0.543183	2.338075	-0.117674
6	6	0	0.438972	3.322097	-0.154140
7	1	0	2.563213	3.704244	-0.126826
8	1	0	3.166453	1.343649	0.017668
9	1	0	-1.592327	2.624561	-0.162927
10	1	0	0.151850	4.370706	-0.226771
11	6	0	1.630514	-0.866407	0.184087
12	6	0	3.125658	-1.060588	-0.065638
13	1	0	3.724302	-0.619624	0.740587
14	1	0	3.333593	-2.135383	-0.085344
15	1	0	3.428125	-0.631498	-1.027634
16	53	0	-1.890839	-0.308209	0.027589
17	6	0	1.252859	-1.427445	1.557519
18	1	0	0.173782	-1.400882	1.728767
19	1	0	1.600259	-2.461628	1.654372
20	1	0	1.745398	-0.819344	2.326474
21	8	0	0.913896	-1.507279	-0.890551
22	9	0	1.129561	-2.908843	-0.770489

Compound 4a

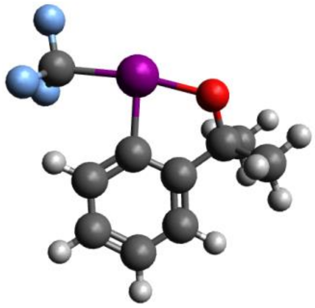

 # nmr=giao wb97xd/genecp geom=connectivity

Center Number	Atomic Number	Atomic Type	Coordinates (Angstroms) X	Y	Z
1	6	0	2.331673	2.863305	0.010524
2	6	0	2.768707	1.542668	0.050712
3	6	0	1.858237	0.481946	-0.003541
4	6	0	0.515555	0.812916	-0.082811
5	6	0	0.038417	2.116618	-0.124436
6	6	0	0.970312	3.151810	-0.081066
7	1	0	3.058450	3.674239	0.047998
8	1	0	3.834596	1.331508	0.126486
9	1	0	-1.020091	2.345678	-0.196727
10	1	0	0.625302	4.184189	-0.121361
11	6	0	2.278674	-0.993207	0.057894
12	6	0	3.438945	-1.268450	-0.905071
13	1	0	4.354648	-0.738263	-0.610853
14	1	0	3.647737	-2.345140	-0.899714
15	1	0	3.163596	-0.971696	-1.924126
16	8	0	1.199030	-1.788247	-0.347460
17	53	0	-0.727188	-0.928139	-0.119087
18	6	0	-2.611170	0.304702	0.095910
19	9	0	-2.901515	1.109672	-0.951632
20	9	0	-3.612759	-0.602173	0.183371
21	9	0	-2.669703	1.069494	1.206833
22	6	0	2.679734	-1.330801	1.504156
23	1	0	1.841732	-1.146064	2.189620
24	1	0	2.948588	-2.392791	1.563788
25	1	0	3.535463	-0.726284	1.835388

Compound 4b

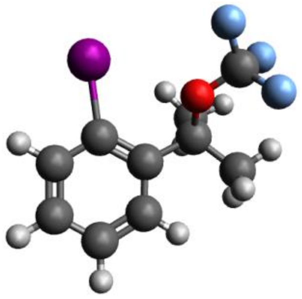

 # nmr=giao wb97xd/genecp geom=connectivity

Center Number	Atomic Number	Atomic Type	Coordinates (Angstroms) X	Y	Z
1	6	0	-2.373737	2.940500	-0.431767
2	6	0	-1.061176	2.657400	-0.067906
3	6	0	-0.589076	1.350172	0.113458
4	6	0	-1.517445	0.309436	-0.092953
5	6	0	-2.835438	0.588406	-0.464855
6	6	0	-3.269494	1.899219	-0.633803
7	1	0	-2.686773	3.975801	-0.560829
8	1	0	-0.385001	3.495216	0.075376
9	1	0	-3.533491	-0.231468	-0.622418
10	1	0	-4.302252	2.094658	-0.920619
11	6	0	0.864313	1.145141	0.572059
12	6	0	1.716619	2.409094	0.468266
13	1	0	1.356941	3.158618	1.181858
14	1	0	2.751751	2.181312	0.740328
15	1	0	1.704296	2.829813	-0.542789
16	53	0	-1.127315	-1.772975	0.106123
17	6	0	0.889641	0.632018	2.016367
18	1	0	0.416036	-0.349143	2.104295
19	1	0	1.915049	0.565470	2.392098
20	1	0	0.331951	1.342133	2.639446
21	8	0	1.360411	0.132151	-0.364438
22	6	0	2.646527	-0.185857	-0.493002
23	9	0	2.725000	-1.350356	-1.143463
24	9	0	3.352493	0.717723	-1.214865
25	9	0	3.308231	-0.325715	0.679332

Compound 4c

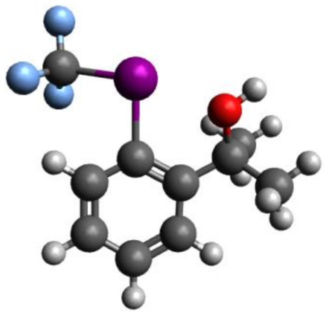

----
nmr=giao wb97xd/genecp geom=connectivity

Center Number	Atomic Number	Atomic Type	Coordinates (Angstroms) X	Y	Z
1	6	0	2.296788	2.871764	0.030481
2	6	0	2.760408	1.563459	0.126257
3	6	0	1.900409	0.463427	0.026096
4	6	0	0.553504	0.773913	-0.160232
5	6	0	0.051581	2.062618	-0.291827
6	6	0	0.946298	3.124812	-0.187810
7	1	0	3.000932	3.697954	0.112974
8	1	0	3.823633	1.391948	0.279169
9	1	0	-0.998222	2.266113	-0.479871
10	1	0	0.577019	4.144027	-0.287068
11	6	0	2.440728	-0.957705	0.153903
12	6	0	3.823443	-1.123512	-0.470752
13	1	0	4.593647	-0.612570	0.117735
14	1	0	4.087352	-2.189175	-0.484031
15	1	0	3.839477	-0.743204	-1.497934
16	53	0	-0.849435	-0.853665	-0.278503
17	6	0	-2.677703	0.277562	0.258027
18	9	0	-3.042317	1.096945	-0.716147
19	9	0	-3.605886	-0.650104	0.438147
20	9	0	-2.484380	0.959971	1.372405
21	6	0	2.429897	-1.398573	1.619574
22	1	0	1.426243	-1.324595	2.056705
23	1	0	2.780529	-2.435507	1.710219
24	1	0	3.102757	-0.761800	2.205749
25	8	0	1.511312	-1.762761	-0.615700
26	1	0	1.737344	-2.694741	-0.519354

Compound 5a

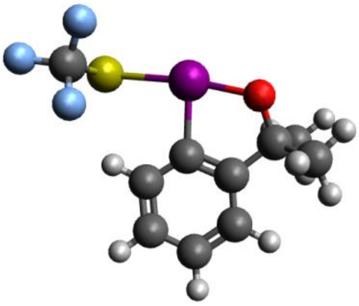

---

nmr=giao wb97xd/genecp geom=connectivity

Center Number	Atomic Number	Atomic Type	Coordinates (Angstroms) X	Y	Z
1	6	0	2.403150	3.054912	0.021710
2	6	0	2.973594	1.798893	-0.168141
3	6	0	2.191325	0.641211	-0.107917
4	6	0	0.842424	0.816470	0.156850
5	6	0	0.234656	2.043887	0.358339
6	6	0	1.038531	3.180412	0.282119
7	1	0	3.028831	3.945314	-0.034185
8	1	0	4.041817	1.714868	-0.364997
9	1	0	-0.829806	2.118722	0.566784
10	1	0	0.591314	4.163159	0.425685
11	6	0	2.750006	-0.773148	-0.269283
12	6	0	3.678392	-0.866508	-1.482992
13	1	0	4.595680	-0.281059	-1.336118
14	1	0	3.960375	-1.915750	-1.631112
15	1	0	3.164828	-0.510450	-2.383558
16	8	0	1.675575	-1.658816	-0.509662
17	53	0	-0.180500	-1.057367	0.237078
18	6	0	3.487955	-1.171080	1.017924
19	1	0	2.803407	-1.151628	1.876477
20	1	0	3.884110	-2.188395	0.910141
21	1	0	4.319306	-0.483993	1.225445
22	16	0	-2.348836	-0.016799	1.143643
23	6	0	-3.226548	0.283384	-0.406386
24	9	0	-2.587021	1.151462	-1.223340
25	9	0	-4.435582	0.808813	-0.140061
26	9	0	-3.428228	-0.836012	-1.131491

Compound 5b

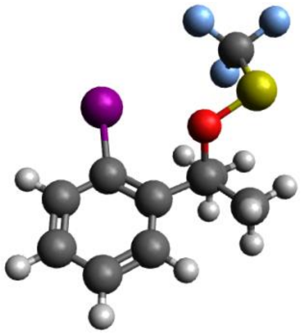

----
nmr=giao wb97xd/genecp geom=connectivity

Center Number	Atomic Number	Atomic Type	Coordinates (Angstroms) X	Y	Z
1	6	0	-3.980575	1.591259	-0.295567
2	6	0	-2.671085	2.001138	-0.067562
3	6	0	-1.601497	1.101594	0.054064
4	6	0	-1.916697	-0.266865	-0.065026
5	6	0	-3.229499	-0.684791	-0.303048
6	6	0	-4.263907	0.237528	-0.418735
7	1	0	-4.771719	2.335208	-0.380705
8	1	0	-2.486225	3.067760	0.018025
9	1	0	-3.444345	-1.747465	-0.398508
10	1	0	-5.280504	-0.108855	-0.602382
11	6	0	-0.198587	1.659094	0.350272
12	6	0	-0.106937	3.173528	0.161016
13	1	0	-0.708770	3.703181	0.908065
14	1	0	0.931983	3.489513	0.309683
15	1	0	-0.426764	3.471382	-0.844571
16	53	0	-0.537329	-1.874215	0.086527
17	6	0	0.243328	1.299428	1.768174
18	1	0	0.251166	0.219425	1.934630
19	1	0	1.245593	1.696846	1.967944
20	1	0	-0.456708	1.757621	2.477808
21	8	0	0.637595	1.024418	-0.677851
22	16	0	2.247436	1.456732	-0.768760
23	6	0	3.082358	-0.023411	-0.111163
24	9	0	2.854785	-1.128926	-0.827979
25	9	0	4.396908	0.265299	-0.191267
26	9	0	2.795371	-0.312514	1.165077

Compound 6a

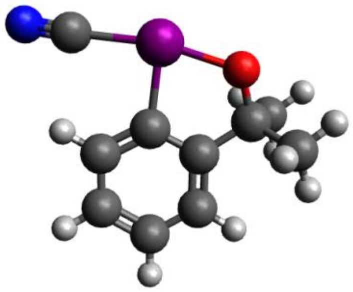

----

nmr=giao wb97xd/genecp geom=connectivity

Center Number	Atomic Number	Atomic Type	Coordinates (Angstroms) X	Y	Z
1	6	0	2.094378	2.771719	-0.008583
2	6	0	2.444375	1.424179	0.012530
3	6	0	1.462291	0.428567	-0.017060
4	6	0	0.147258	0.859462	-0.057720
5	6	0	-0.249684	2.187207	-0.073314
6	6	0	0.753492	3.154894	-0.052853
7	1	0	2.874588	3.531869	0.011019
8	1	0	3.495515	1.141341	0.056200
9	1	0	-1.300302	2.470745	-0.099354
10	1	0	0.480918	4.209226	-0.071836
11	6	0	1.769386	-1.072372	0.045564
12	6	0	2.869915	-1.450237	-0.949196
13	1	0	3.829689	-0.983419	-0.691771
14	1	0	3.001837	-2.538817	-0.932462
15	1	0	2.584989	-1.147249	-1.963586
16	8	0	0.612833	-1.788838	-0.323068
17	53	0	-1.200880	-0.793029	-0.073635
18	6	0	2.178822	-1.434597	1.481667
19	1	0	1.378298	-1.177782	2.188142
20	1	0	2.364603	-2.513915	1.543325
21	1	0	3.088577	-0.897871	1.783078
22	7	0	-3.802799	1.323921	0.229470
23	6	0	-2.876092	0.627788	0.128661

Compound 6b

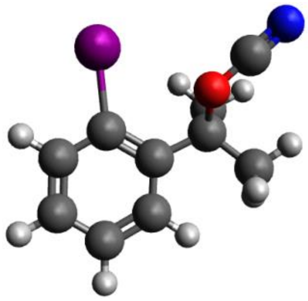

\# nmr=giao wb97xd/genecp geom=connectivity

Center Number	Atomic Number	Atomic Type	Coordinates (Angstroms) X	Y	Z
1	6	0	-2.353523	2.670797	-0.191661
2	6	0	-0.975550	2.551295	-0.043575
3	6	0	-0.329862	1.309525	0.029909
4	6	0	-1.143523	0.161781	-0.049211
5	6	0	-2.527004	0.277218	-0.208040
6	6	0	-3.135627	1.526231	-0.278419
7	1	0	-2.808516	3.658871	-0.245185
8	1	0	-0.390388	3.465100	0.014753
9	1	0	-3.136426	-0.622219	-0.274608
10	1	0	-4.215922	1.595564	-0.401575
11	6	0	1.182396	1.288615	0.254575
12	6	0	1.884744	2.610884	-0.035251
13	1	0	1.629222	3.362621	0.719833
14	1	0	2.969097	2.452776	0.014152
15	1	0	1.629049	2.989980	-1.030969
16	53	0	-0.460912	-1.849253	0.053280
17	6	0	1.533933	0.798928	1.657338
18	1	0	1.088100	-0.175166	1.876011
19	1	0	2.621655	0.729629	1.782702
20	1	0	1.146246	1.529002	2.378139
21	8	0	1.685629	0.334399	-0.776057
22	6	0	2.910450	-0.030808	-0.671658
23	7	0	4.018939	-0.376822	-0.604669

1-(Pentafluoroethyl)-3-methyl-3-isopropyl-1,2-benziodoxole

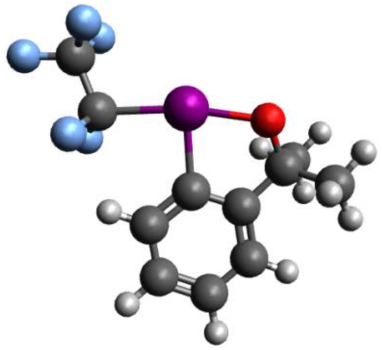

---

nmr=giao wb97xd/genecp geom=connectivity

Center Number	Atomic Number	Atomic Type	Coordinates (Angstroms) X	Y	Z
1	6	0	3.178865	2.678629	0.080599
2	6	0	3.477743	1.320454	0.129656
3	6	0	2.468379	0.356786	0.032078
4	6	0	1.169310	0.822816	-0.095693
5	6	0	0.829191	2.168017	-0.151514
6	6	0	1.858818	3.103000	-0.064859
7	1	0	3.980964	3.412657	0.151616
8	1	0	4.513031	1.002082	0.244031
9	1	0	-0.194842	2.505534	-0.266773
10	1	0	1.622056	4.164895	-0.115521
11	6	0	2.734495	-1.153771	0.095802
12	6	0	3.930384	-1.537035	-0.782175
13	1	0	4.871049	-1.115637	-0.403175
14	1	0	4.022334	-2.629811	-0.788307
15	1	0	3.770574	-1.193839	-1.811124
16	8	0	1.612950	-1.826518	-0.408188
17	53	0	-0.229302	-0.797786	-0.201031
18	6	0	2.988884	-1.546649	1.561195
19	1	0	2.122435	-1.290870	2.186074
20	1	0	3.151634	-2.629949	1.620795
21	1	0	3.869498	-1.030271	1.967000
22	6	0	-1.996325	0.645865	0.022909
23	6	0	-3.315348	-0.142766	0.134301
24	9	0	-2.127439	1.490356	-1.042270
25	9	0	-1.893811	1.418164	1.140563
26	9	0	-4.375475	0.661543	0.247551
27	9	0	-3.480852	-0.909044	-0.957155
28	9	0	-3.280836	-0.949023	1.207057

1-Trifluoro-3-methyl-3-isopropyl-1,2-benziodoxole

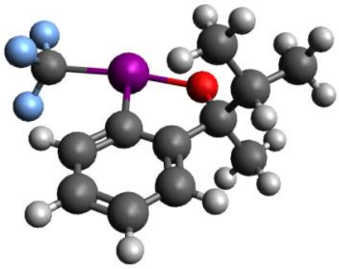

---

nmr=giao wb97xd/genecp geom=connectivity

Center Number	Atomic Number	Atomic Type	Coordinates (Angstroms) X	Y	Z
1	6	0	-1.339361	3.423613	0.056506
2	6	0	-2.046228	2.245214	-0.156737
3	6	0	-1.388411	1.012299	-0.239453
4	6	0	-0.011436	1.031012	-0.101109
5	6	0	0.732060	2.185359	0.113103
6	6	0	0.048943	3.396275	0.191539
7	1	0	-1.872710	4.371759	0.117466
8	1	0	-3.130107	2.279811	-0.260611
9	1	0	1.811949	2.169709	0.218583
10	1	0	0.606341	4.317202	0.356676
11	6	0	-2.136896	-0.308404	-0.466186
12	6	0	-3.058102	-0.584947	0.761644
13	1	0	-3.744469	0.272865	0.847753
14	8	0	-1.211215	-1.346307	-0.621150
15	53	0	0.835938	-0.925736	-0.241968
16	6	0	2.919446	-0.163120	0.170706
17	9	0	3.086591	0.429909	1.372481
18	9	0	3.701313	-1.268005	0.161150
19	9	0	3.419537	0.681835	-0.757985
20	6	0	-2.947110	-0.197182	-1.767982
21	1	0	-2.294746	0.142271	-2.581577
22	1	0	-3.341244	-1.183083	-2.035267
23	1	0	-3.786998	0.504587	-1.671532
24	6	0	-2.253118	-0.683560	2.058680
25	1	0	-2.925833	-0.836146	2.912540
26	1	0	-1.565397	-1.538963	2.017088
27	1	0	-1.669384	0.225624	2.254692
28	6	0	-3.894645	-1.849717	0.559932
29	1	0	-3.241977	-2.702487	0.330780
30	1	0	-4.455290	-2.082117	1.475386
31	1	0	-4.622958	-1.741236	-0.253137

1-Chloro-3-ethyl-3-methyl-1,2-benziodoxole

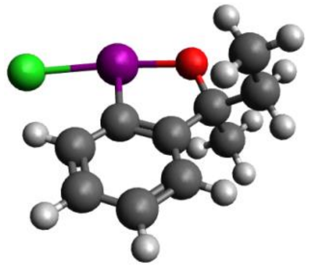

 # nmr=giao wb97xd/genecp geom=connectivity

Center Number	Atomic Number	Atomic Type	Coordinates (Angstroms) X	Y	Z
1	6	0	1.800973	2.984013	0.125951
2	6	0	2.237340	1.672002	0.293083
3	6	0	1.334888	0.606974	0.227500
4	6	0	0.005766	0.930632	0.003355
5	6	0	-0.475438	2.218994	-0.159511
6	6	0	0.452176	3.258783	-0.101402
7	1	0	2.520806	3.800536	0.173246
8	1	0	3.293174	1.471416	0.472645
9	1	0	-1.534518	2.406228	-0.323330
10	1	0	0.114086	4.285731	-0.233876
11	6	0	1.723177	-0.854891	0.441797
12	6	0	2.977022	-1.240366	-0.361706
13	1	0	3.844034	-0.704349	0.052108
14	1	0	3.152213	-2.307988	-0.170259
15	8	0	0.670936	-1.678966	-0.034290
16	53	0	-1.203668	-0.818555	-0.026805
17	6	0	1.941762	-1.099209	1.940730
18	1	0	1.032891	-0.855960	2.507102
19	1	0	2.183999	-2.156047	2.106998
20	1	0	2.761899	-0.478160	2.324810
21	17	0	-3.328147	0.545062	-0.103662
22	6	0	2.859567	-0.994596	-1.862112
23	1	0	3.752811	-1.363607	-2.381525
24	1	0	1.985419	-1.517723	-2.268058
25	1	0	2.755210	0.074980	-2.088759

1-Chloro-3-methyl-3-propyl-1,2-benziodoxole

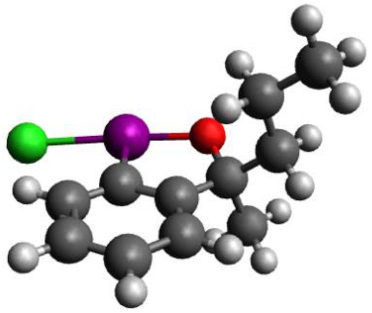

----

nmr=giao wb97xd/genecp geom=connectivity

Center Number	Atomic Number	Atomic Type	Coordinates (Angstroms) X	Y	Z
1	6	0	1.085030	3.279100	0.139871
2	6	0	1.668646	2.070612	0.511695
3	6	0	0.944407	0.876944	0.442243
4	6	0	-0.366123	0.971350	0.001627
5	6	0	-0.990672	2.149856	-0.371243
6	6	0	-0.237748	3.321387	-0.302130
7	1	0	1.666875	4.198613	0.195756
8	1	0	2.701833	2.052401	0.857557
9	1	0	-2.027590	2.154770	-0.700507
10	1	0	-0.691681	4.267566	-0.593676
11	6	0	1.497539	-0.482020	0.869995
12	6	0	2.893553	-0.739118	0.279964
13	1	0	3.606125	-0.026601	0.724450
14	1	0	3.202429	-1.738069	0.621349
15	8	0	0.646738	-1.502361	0.368241
16	53	0	-1.302480	-0.934786	0.001371
17	6	0	1.537258	-0.544119	2.402813
18	1	0	0.534332	-0.390850	2.822663
19	1	0	1.902396	-1.529022	2.718911
20	1	0	2.201447	0.230255	2.809238
21	17	0	-3.557034	0.066973	-0.546893
22	6	0	2.964437	-0.672938	-1.242861
23	1	0	2.237754	-1.382982	-1.659127
24	1	0	2.660561	0.327281	-1.584806
25	6	0	4.362724	-0.986755	-1.768722
26	1	0	4.680360	-1.995736	-1.469076
27	1	0	4.394665	-0.936323	-2.864937
28	1	0	5.103307	-0.273067	-1.378704

1-Chloro-3-methyl-3-isopropyl-1,2-benziodoxole

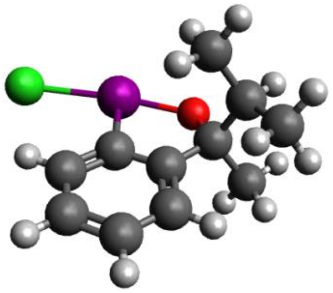

\# nmr=giao wb97xd/genecp geom=connectivity

Center Number	Atomic Number	Atomic Type	Coordinates (Angstroms) X	Y	Z
1	6	0	-1.475654	3.134394	-0.105786
2	6	0	-1.987710	1.859169	-0.332504
3	6	0	-1.154471	0.734958	-0.315745
4	6	0	0.193724	0.975224	-0.088753
5	6	0	0.746978	2.223484	0.141889
6	6	0	-0.115305	3.319139	0.136656
7	1	0	-2.146362	3.992868	-0.123131
8	1	0	-3.050664	1.736568	-0.529251
9	1	0	1.814688	2.339844	0.314606
10	1	0	0.285032	4.316995	0.310920
11	6	0	-1.648548	-0.694436	-0.531374
12	6	0	-2.442647	-1.233724	0.698052
13	1	0	-2.643220	-2.282429	0.431050
14	8	0	-0.525581	-1.543854	-0.736844
15	53	0	1.327064	-0.817200	-0.191184
16	6	0	-2.474157	-0.781451	-1.819877
17	1	0	-1.840536	-0.511511	-2.672387
18	1	0	-2.826893	-1.811168	-1.957043
19	1	0	-3.342889	-0.113168	-1.807505
20	17	0	3.458846	0.369321	0.453773
21	6	0	-1.610957	-1.230438	1.981955
22	1	0	-2.192149	-1.665159	2.805591
23	1	0	-0.696751	-1.824919	1.876776
24	1	0	-1.335743	-0.207216	2.276952
25	6	0	-3.787995	-0.547514	0.949791
26	1	0	-4.336704	-1.097696	1.725523
27	1	0	-3.652813	0.478282	1.318481
28	1	0	-4.424695	-0.520156	0.056966

1-Chloro-3-methyl-3-isobutyl-1,2-benziodoxole

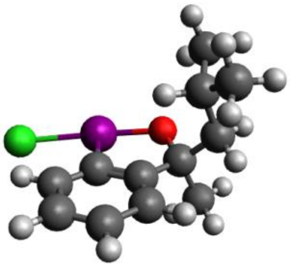

---

nmr=giao wb97xd/genecp geom=connectivity

Center Number	Atomic Number	Atomic Type	Coordinates (Angstroms) X	Y	Z
1	6	0	0.837252	3.381128	-0.013248
2	6	0	1.434842	2.235236	0.505076
3	6	0	0.755030	1.013475	0.507533
4	6	0	-0.527371	1.017184	-0.017955
5	6	0	-1.164387	2.131549	-0.538322
6	6	0	-0.455661	3.332116	-0.535483
7	1	0	1.384763	4.323080	-0.009233
8	1	0	2.443523	2.289713	0.912943
9	1	0	-2.176951	2.065571	-0.930846
10	1	0	-0.919787	4.229302	-0.942823
11	6	0	1.331264	-0.277991	1.088886
12	8	0	0.524700	-1.370142	0.676023
13	53	0	-1.404178	-0.913540	0.100771
14	6	0	1.321394	-0.184248	2.620503
15	1	0	0.298920	-0.025557	2.987627
16	1	0	1.704640	-1.119781	3.045934
17	1	0	1.945978	0.648789	2.969432
18	17	0	-3.635735	-0.054581	-0.709857
19	6	0	2.891241	-0.639267	-0.962909
20	1	0	2.082351	-0.051032	-1.422785
21	6	0	2.756454	-0.528417	0.567053
22	1	0	3.123911	-1.449612	1.042531
23	1	0	3.397292	0.281933	0.944498
24	6	0	4.219035	-0.040043	-1.430488
25	1	0	5.067237	-0.551542	-0.950169
26	1	0	4.338884	-0.143661	-2.517560
27	1	0	4.284325	1.028842	-1.182071
28	6	0	2.749636	-2.084437	-1.445524
29	1	0	3.581568	-2.697280	-1.065984
30	1	0	1.811854	-2.525371	-1.090717
31	1	0	2.770459	-2.134500	-2.542992
